# Optimization of an Ultrasound-Assisted Extraction Method for the Analysis of Major Anthocyanin Content in *Erica australis* Flowers

**DOI:** 10.3390/molecules26102884

**Published:** 2021-05-13

**Authors:** Ceferino Carrera, María José Aliaño-González, Jaime Rodríguez-López, Marta Ferreiro-González, Fernando Ojeda-Copete, Gerardo F. Barbero, Miguel Palma

**Affiliations:** 1 Department of Analytical Chemistry, Faculty of Sciences, Agrifood Campus of International Excellence (ceiA3), IVAGRO, University of Cadiz, Puerto Real, 11510 Cadiz, Spain; ceferino.carrera@uca.es (C.C.); mariajose.alianogonzalez@alum.uca.es (M.J.A.-G.); marta.ferreiro@uca.es (M.F.-G.); miguel.palma@uca.es (M.P.); 2 Department of Biology, Faculty of Marine and Environmental Sciences, University of Cadiz, Puerto Real, 11510 Cadiz, Spain; jaime.rodriguezlopez@alum.uca.es (J.R.-L.); fernando.ojeda@uca.es (F.O.-C.)

**Keywords:** flower anthocyanins, Mediterranean heather, ultrasound-assisted extraction, optimization, Box-Behnken design, HPLC-UV-vis

## Abstract

*Erica australis* plants have been used in infusions and folk medicine for years for its diuretic and antiseptic properties and even for the treatment of infections. In addition, a recently published thorough study on this species has demonstrated its antioxidant, antibiotic, anti-inflammatory, anticarcinogenic and even antitumoral activities. These properties have been associated with the high content of anthocyanins in *E. australis* leaves and flowers. The aim of the present research is to optimize an ultrasound-assisted extraction methodology for the recovery of the anthocyanins present in *E. australis* flowers. For that purpose, a Box Behnken design with response surface methodology was employed, and the influence of four variables at different values was determined: namely, the composition of the extraction solvents (0–50% MeOH in water), the pH level of those solvents (3–7), the extraction temperature (10–70 °C), and the sample:solvent ratio (0.5 g:10 mL–0.5 g:20 mL). UHPLC-UV-vis has been employed to quantify the two major anthocyanins detected in the samples. The extraction optimum conditions for 0.5 g samples were: 20 mL of solvent (50% MeOH:H_2_O) at 5 pH, with a 15 min extraction time at 70 °C. A precision study was performed and the intra-day and inter-day relative standard deviations (RSDs) obtained were 3.31% and 3.52%, respectively. The developed methodology has been successfully applied to other *Erica* species to validate the suitability of the method for anthocyanin extraction.

## 1. Introduction

The Mediterranean heather *Erica australis* L. is a medium-sized, evergreen shrub of the *Ericaceae* family. Plants in this species can reach up to 2 m high with profuse flowering of distinctive 6.0–8.5 mm long, pink to rose-purple flowers. They can be mainly found in Mediterranean heathlands of the W Iberian Peninsula and NW tip of Africa [1,2].

This species readily recovers from fire, both by the resprouting of surviving plants and by seedling recruitment from a permanent soil seed-bank [3,4,5]. For this reason, their use in areas which have recently suffered the effects of wildfire or that are prone to suffer such effects has notably increased [4]. On the other hand, *E. australis* has been extensively used as infusions, especially in Moroccan and Turkish folk medicine because of their demonstrated diuretic and antiseptic properties, and even to directly treat infected wounds [6,7].

This growing number of applications has increased the interest of multiple researchers for the plant composition and how it influences its applications. Some extracts from this plant have exhibited important health promoting properties such as antioxidant, antibiotic, anti-inflammatory, anticarcinogenic and antitumoral [8]. In addition, it has also been proven that it can increase the metabolic activity in the fibroblast proliferation that is closely related to tissue regeneration [9]. These are the reasons why they have been used for the treatment of different diseases, including tumoral processes [10], prostate, kidney or bladder disorders [11], or even for the treatment of hyperlipidemia, which has an important impact on cardiovascular pathologies [12]. In addition, its use in the cosmetic industry has also grown considerably because of its antioxidant activity as well as for its color and other physical properties [13]. Finally, its flowers are also and increasingly being used in the agri-food industry as an accompaniment in multiple gourmet plates. The consumption of *E. australis* flowers as part of our diet is meant to promote significant health benefits.

An in depth study on the composition of *E. australis* has demonstrated its substantial concentrations of amino acids and phenolic compounds and, particularly, of anthocyanins, which are responsible for many of the biological properties of this plant, and can be found in greater concentrations in its leaves and flowers [14,15,16]. Anthocyanins are a subcategory of phenolic compounds [17,18] that act as natural pigments to provide different vegetables and fruits such as sloes, myrtle, maqui berry, açai, blueberry, *Camellia sinensis* flowers, *Primula veris*, etc. with their characteristic red or blue color [19,20,21,22,23,24]. These compounds have been associated with specific health promoting properties like antioxidant, anti-inflammatory, anticarcinogenic, or with beneficial effects on the treatment of obesity, microcirculation, cardiovascular or neurodegenerative disorders [25,26,27,28]. In addition, they have exhibited important vasodilatation capacity with pain relief effects. For all these reasons, relying on efficient methodologies for the extraction and analysis of anthocyanins from *E. australis*, while allowing their analysis and control, would be really important for decisive medical, cosmetic, pharmaceutical applications, and even for their use in the food industry as a natural colorant.

Even though the benefits and properties of *E. australis* have been extensively confirmed, there are scarce research studies investigating in depth the use of innovative extraction techniques for the analysis of anthocyanins in *Erica* plants, being solid-liquid, microwave hydrodiffusion and gravity extractions the most frequently used methods [8,9,12,13]. Ultrasound-assisted extraction (UAE) is a technique based on the phenomenon of cavitation caused by the ultrasound energy and its mechanical effect on the plant matrix, which favors the mass transfer from the sample into the solvent, thus achieving an efficient extraction of the desirable compounds from different matrices [29]. In addition, the application of high temperatures increases the number and size of the bubbles that are generated by the cavitation phenomenon and further improve the efficiency of the extraction method in most cases [30].

UAE represents an important improvement with respect to other extraction techniques, since it is easy-to-use, economical, rapid and only requires low amounts of solvents. Other extraction techniques (pressure liquid extraction (PLE) or supercritical fluid extraction (SFE)) have some of these advantages such as speed in extraction or low consumption of solvents, but they are more expensive techniques and with less availability in most analytical laboratories [31,32]. Furthermore, it does not require any complex maintenance operations and has been extensively used all over the world, which means that we are all quite familiarized with its characteristics and application methods [33]. In conclusion, UAE employs simple and relatively cheap instrumentation, presents low energy consumption, the time of analysis is quite short, does not require the application of pressure, and high temperatures are not needed. Furthermore, the phenomenon of cavitation produced by the effect of ultrasound in the extracting medium, favors cell rupture and the transmission of the analytes of interest within the solution. In fact, it has already been used for the extraction of anthocyanins from numerous matrices such as onion (*Allium cepa*), lavender (*Lavandula* spp.), blackberries (*Rubus* spp.), pepper (*Capsicum* spp.), açai (*Euterpe oleracea*), purple potato (*Solanum tuberosum*), blackthorn (*Prunus spinosa*), breadnut (*Brosimum alicastrum*), etc. [34,35,36,37,38,39,40,41,42].

Multiple methodologies have been employed for the separation and quantification of anthocyanins in food matrixes as liquid chromatography with ultraviolet/visible detection or liquid chromatography-mass spectrometry (LC-UV-vis and LC-MS) [24,43]. However, the technique most used for the analysis of anthocyanins in the *Erica* genus has been high-performance liquid chromatography with diode-array detection (HPLC-DAD) [15,44,45]. In this study, ultra-high-performance liquid chromatography with diode-array detection (UHPLC-DAD) has been used for its ability to separate compounds with high chromatographic resolution, due to the use of particles sizes smaller than 2 µm, which produces an increase in the working pressure and a shortening of the analysis times.

The present study intends to develop and optimize a methodology based on ultrasound-assisted for the extraction of anthocyanins from *E. australis*. The different variables involved in such method have been optimized based on a Box-Behnken design (BBD) combined with response surface methodology (RSM). The final aim of this study is to propose a novel extraction method of anthocyanins using UAE that allows to determine the anthocyanin content in *Erica australis* L. flower and other *Erica* species for different purposes.

## 2. Materials and Methods

### 2.1. Biological Material

*Erica australis* flowers (fresh) were collected in March 2019 from Los Barrios (Cadiz, Spain). The dry *Erica australis*, *Erica arborea* L. and *Erica scoparia* L. flowers were acquired from Zulueta Corporación (Tudela, Spain). All the samples were crushed with a ZM200 knife mill (Retsch GmbH, Haan, Germany) and stored in a freezer at −20 °C until further extraction.

### 2.2. Chemical and Solvents

Methanol (Fisher Chemical, Loughborough, UK) of HPLC grade and Milli-Q water, obtained from a Milli-Q water purification system (Millipore, Bedford, MA, USA) were selected as the extraction solvents. The liquid-liquid mixtures were carried out under the required percentage for each extraction and pH values were adjusted with 1 M solutions prepared with HCl (37%—ACS reagent) and NaOH (99%—ACS reagent) (Panreac, Barcelona, Spain).

For the chromatographic separation, methanol (Fisher Scientific, Waltham, MA, USA), and formic acid (Panreac, Barcelona, Spain) both HPLC grade were utilized.

Finally, cyanidin chloride (≥95% purity, Sigma-Aldrich Chemical Co., St. Louis, MO, USA) was employed as the commercial standard for the quantification of the extracted anthocyanins.

### 2.3. Ultrasound-Assisted Extraction

#### 2.3.1. Ultrasound-Assisted Extraction Equipment

The ultrasound-assisted extraction equipment employed was a UP200S sonifier (200 W, 24 kHz) (Dr. Hielscher. GmbH, Berlin, Germany) with a water bath coupled to a temperature controller (FRIGITERM-10, J.P. Selecta, S.A., Barcelona, Spain).

#### 2.3.2. Ultrasound-Assisted Extraction Optimization

Dry *E. australis* flowers were used for the UAE optimization based on a BDD with RSM. This design is one of the most widely used to optimize different processes, since it allows to determine the best values of the selected variables of interest, each variable’s significance and the effects caused by the interactions between the selected variables. In order to generate this type of design, three variable values are selected per factor: (−1) a lower level, (0) an intermediate level, and (1) a higher level. The design is characterized by lacking an embedded factorial or fractional factorial design and by not presenting any axial points. Instead, it generates a more spherical arrangement of the design points. Thanks to these characteristics, any experiment to be run under extreme or even unfeasible conditions that would cause a serious degradation of the samples or would imply unaffordable costs are excluded [46]. Four independents factors were selected to be investigated within the following ranges: composition of the extraction solvent (%MeOH in water, with values from 0 to 50%), extraction temperature (with values from 10 to 70 °C), pH of the solvent (with values from 3 to 7), sample to solvent ratio (values from 10 to 20 (0.5 g:10 mL–0.5 g:20 mL)). The extraction time was set at 10 min for all experiments.

The percentage of MeOH, the extraction temperature, and the solvent ratio were determined based on previous investigations carried out in the research group and bibliograpy [32,35,36]. Regarding the pH of the solvent, in aqueous solution, anthocyanins undergo structural re-arrangements in response to changes in pH in four molecular structures: quinoidal base (blue), flavylium cation (red), carbinol (colorless) and chalcone (yellowish) forms. Anthocyanins are stable in acidic solutions (pH 1–3) where they exist primarily as flavylium cations. At pH > 4, anthocyanins adopt the forms of the carbinol and chalcone [47]. In previous studies, it has been shown that pH values close to 7 are capable of extracting a greater quantity of anthocyanins in hydroalcoholic mixtures [19,21,38,48]. pH values greater than 7 have not been tested, due to the instability of these compounds at basic pH.

The BBD-RSM methodology was based on 27 extractions including three repetitions at the center point to determine the error. All the experiments were randomly performed. The response variable to be studied was the total relative area of the anthocyanins extracted from *E. australis* flowers samples under different conditions. This was calculated as the total sum of the areas corresponding to each one of the two major individual anthocyanins that had been previously identified. A summary of the experimental conditions according to the BBD-RSM and their results can be seen in Table 1.

Once the extractions had been performed under the established experimental conditions, the resulting extracts were double centrifuged for 5 min at 7500 rpm (orbital radius 9.5 cm) and filtered using 0.20-μm nylon syringe filter (Membrane Solutions, Dallas, TX, USA) prior to their analysis for identification and/or quantification.

### 2.4. Identification of Anthocyanins by UHPLC-QToF-MS

The anthocyanins present in the flower samples were identified by Ultra-Performance Liquid Chromatography coupled to a Photodiode Array-Quadrupole-Time-of-Flight Mass Spectrometer, (UHPLC–PDA-QToF–MS) (Xevo G2, Waters Corp., Milford, MA, USA). A 100 × 2.1 mm, particle size of 1.7 μm, reverse-phase C18 analytical column (Acquity UPLC BEH C18, Waters) was used. The solvent A employed for the analysis was formed by water containing 2% formic acid, whereas solvent B was methanol with 2% formic acid. A 0.4 mL/min flow rate was applied. The gradient followed for the identification was 5% B at 0 min; 20% B at 3.30 min; 30% B at 3.86 min; 40% B at 5.05 min; 55% B at 5.35 min; 60% B at 5.64 min; 95% B at 5.94 min; 95% B at 7.50 min. This represents 12-min total analysis time, including the 4 min required for re-equilibration. The solution was ionized by means of an electrospray source in positive ionization mode and a desolvation gas flow of 700 L/h at 500 °C and a capillary cone voltage of 700 V. In addition, the cone gas flow was of 10 L/h at 150 °C source temperature and 20 V with a trap collision energy of 4 eV. The full-scan mode was applied in the range 100–1200 *m*/*z*.

**Figure 1:**
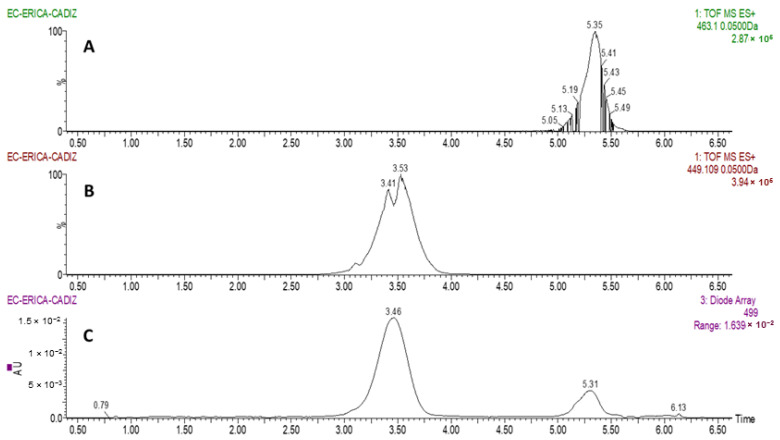
Chromatograms obtained from the analysis of *Erica australis* flowers by UHPLC-PDA-QToF-MS. (**A**): SIM chromatogram at *m*/*z* 463.1 (peonidin 3-*O*-glucoside); (**B**): SIM chromatogram at *m*/*z* 449.1 (cyanidin 3-*O*-glucoside); (**C**): UV-vis chromatogram at *λ* 499 nm for anthocyanins.

Two major anthocyanins were identified in *Erica australis* L. flowers, as can be observed in Figure 1. Based on molecular ion [M]^+^, they were identified as cyanidin 3-*O*-glucoside (*m*/*z* = 449.1085) and peonidin 3-*O*-glucoside (*m*/*z* = 463.1242). The result are in agreement with previous characterization analyses on other *Erica* species, such as *E. coccinea* [45].

### 2.5. Analysis of Anthocyanins by UHPLC-UV-Vis

Once the identification of anthocyanins was completed, UHPLC-UV-Vis was employed for quantification. An Elite UHPLC LaChrom System (Hitachi, Tokyo, Japan) system was used. The equipment comprised an L-2200U autosampler, an L2300 column oven set at 50 °C, two L-2160U pumps, and a UV–Vis detector L-2420U. The analytical column used was a reversed-phase 2.1 × 50 mm and 2.6 µm particle size C18 (Phenomenex Kinetex, CoreShell Technology, Torrance, CA, USA). Two mobile phases were used as follows: acidified water (5% formic acid) as solvent A and methanol as solvent B, operating at a constant flow rate of 0.7 mL/min. The gradient applied was: 15% B (0 min); 20% B (1.50 min); 30% B (3.30 min); 40% B (4.80 min); 55% B (5.40 min); 60% B (5.90 min); 95% B (6.60 min); 95% B (9.30 min); 15% B (10 min). Anthocyanins were measured at 520 nm.

As it has been previously mentioned, cyanidin chloride was the standard selected for anthocyanins quantification according to the following calibration curve “y = 300,568.88x − 28462.43” and a determination coefficient of R^2^ = 0.9999.

The Shapiro-Wilk test was employed to evaluate the normal distribution of residuals, obtaining a W value of 0.8514 (very close to 1) and a *p*-value of 0.803 (above 0.05), which confirms the normal distribution of the residuals.

The Limit of Detection (LOD) was 0.198 mg/L whereas the Limit of Quantification (LOQ) was 0.662 mg/L based on our previous studies [21]. The cyanidin chloride calibration curve was used to quantified the two major anthocyanins present in *Erica australis* L. flower extracts, assuming that the absorbance of the different anthocyanins were similar while taking into account their individual molecular weight. Each analysis was performed in triplicate.

### 2.6. Optimization Study

Once that the 27 extractions for the BBD-RSM were completed and the total anthocyanins content was determined, a second-order polynomial equation, where all the variables were considered, was applied as follows:

Y = *β*_0_ + *β*_1_X_1_ + *β*_2_X_2_ + *β*_3_X_3_ + *β*_4_X_4_ + *β*_12_X_1_X_2_ + *β*_13_X_1_X_3_ + *β*_14_X_1_X_4_ + *β*_23_X_2_X_3_ + *β*_24_X_2_X_4_ + *β*_34_X_3_X_4_ + *β*_11_X_1_^2^ + *β*_22_X_2_^2^ + *β*_33_X_3_^2^ + *β*_44_X_4_^2^

where Y is the aforesaid response, *β*_0_ corresponds to the ordinate, X_1_ (%MeOH in the solvent), X_2_ (pH solvent), X_3_ (extraction temperature), and X_4_ (sample to solvent ratio) are the independent variables. Finally, *β*_i_ represents the linear coefficients; *β*_ij_ the cross-product coefficients, and *β*_ii_ indicates the quadratic coefficients.

Minitab v10.0 software (Minitab LLC, State College, PA, USA) was employed to perform the optimization analysis based on the effect of each variable on the final variable (total anthocyanins) according to a second-order mathematical model, the surface graphs, the calculation of the optimal levels of the significant variables and a variance analysis.

Lastly, once the variables’ optimum values had been determined, repeatability and intermediate precision studies were carried out. For the repeatability analysis, nine extractions were conducted on the same day and for the intermediate precision study, four extractions were completed on each of three consecutive days (a total of twelve extractions). The coefficient of variation of the total relative areas of the anthocyanins was employed as the variable to determine the repeatability and intermediate precision of the developed method.

### 2.7. Application of the Optimized Method

Once the UAE methodology for the extraction of anthocyanins was optimized for *Erica australis* L. flowers, the developed method was applied to *E. australis* flowers (fresh) collected from Cadiz and to the flower samples from the other *Erica* species (*E. arborea* and *E. scoparia*—dry). The samples were extracted in triplicate under the established optimum conditions. The extracts were then centrifuged as previously described and analyzed by UHPLC-UV-vis.

## 3. Results and Discussion

### 3.1. Optimization of the UAE Method

The optimization of the UAE method was based on BBD-RSM according to four variables: composition of the extraction solvents, pH of the solvent, extraction temperature, and sample to solvent ratio. A BBD had been generated based on a total of 27 experiments (Table 1).

The flower samples were extracted under the required conditions and analyzed by UHPLC-UV-vis (520 nm) and two major anthocyanins were determined. The total anthocyanins (the sum of the relative areas corresponding to each one of the two anthocyanins detected) was established as the response variable.

The real values and the values predicted by the BBD were correlated (Table 1) and an average 5% difference between the measured and predicted responses was obtained. The actual differences ranged between 0% and up to 19%. The R-Squared statistic indicates that this model explained 96.21% of the recovery variability. The *p*-value for lack-of-fit in the ANOVA table (0.375) is greater than 0.05, which means that the model is suitable for its intended purpose. Therefore, the model can be used to optimize the extraction conditions for the recovery of anthocyanins.

The BBD-RSM was then applied and the *p*-values were calculated according to the *t*-test using Minitab software at 95% confidence level, this means that the variables with *p*-values below 0.05 were considered as influential (Table 2).

**Figure 2:**
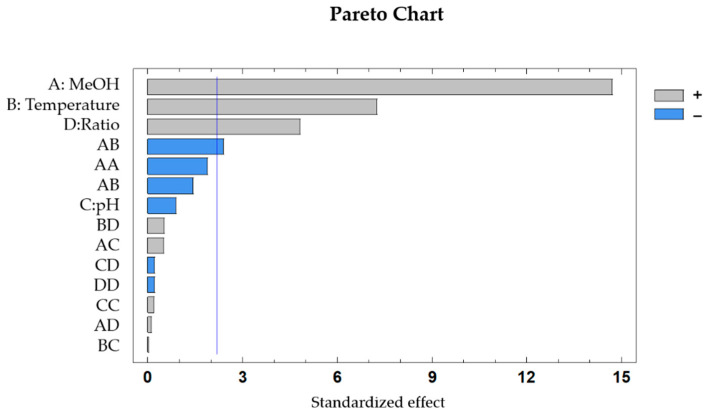
Standardized Pareto chart for the extraction of anthocyanins in *Erica auntralis* flowers (*p* < 0.05). A: %MeOH in the solvent; B: Extraction temperature; C: pH of the extraction solvent; D: Sample to solvent ratio.

The influence of each variable was graphically represented in a Pareto Chart (Figure 2) and as can be observed, the percentage of methanol in the solvent, the extraction temperature and the mass to solvent ratio have significant effects on the recovery of anthocyanins, in these cases with a positive influence in the total anthocyanins recovered. On the other hand, only one interaction presents a significant effect on the recovery of anthocyanins, which is the interaction between solvent composition (%MeOH) and extraction temperature (*p*-value = 0.034), with a negative effect on the response variable.

The coefficients for each of the variables were obtained and replaced, generating the second order polynomial equation for the optimization of the extraction of anthocyanin in *E. australis* flowers:

Y = 563,195 + 187,935.0X_1_ + 92,833.1X_2_ − 11,466.6X_3_ + 61,623.7X_4_ − 52,938.2X_1_X_2_ + 10,870.0X_1_X_3_ + 2441.3X_1_X_4_ + 415.0X_2_X_3_ + 11,409.0X_2_X_4_ − 4524.7X_3_X_4_ − 36,081.8X_1_^2^ − 27,331.9X_2_^2^ + 3734.3X_3_^2^ − 3877.5X_4_^2^

**Figure 3:**
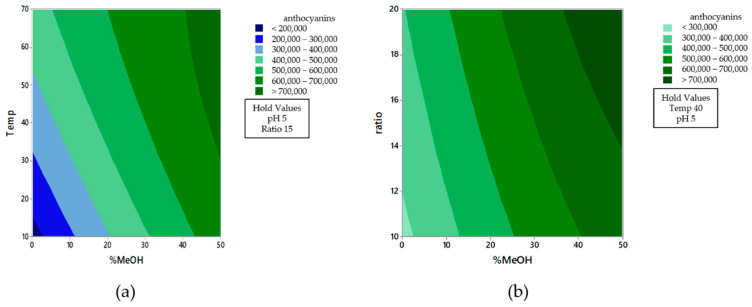
Contour plots of the effect from %MeOH vs. temperature (**a**) and %MeOH vs. ratio (**b**) on the recovery of anthocyanins.

Figure 3 shows the contour plots of the effect from %MeOH vs. Temperature (Figure 3a.) and %MeOH vs. ratio (Figure 3b.), where the interactions either between solvent composition and ratio or between solvent composition and extraction temperature are both more apparent when the methanol percentage in the extraction solvent is low. Therefore, the three extraction variables that affect the recovery of anthocyanins can be separately optimized.

The optimal extraction conditions obtained were 70 °C of temperature, 50% MeOH, pH 5 and 20 mL of solvent per 0.5 g of sample. The ratio solvent to solid of the sample should not be increased, as the signal in the chromatographic system would be too low for a reliable determination. Similarly, the temperature should not be above 70 °C because the solvent would start boiling and this would affect the final recovery.

**Figure 4:**
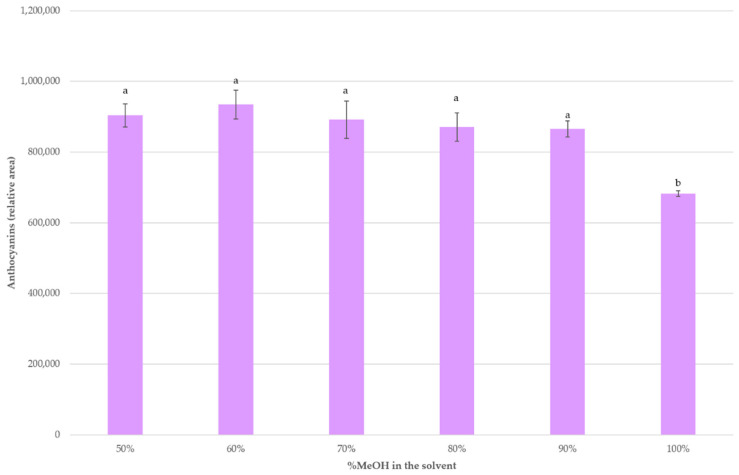
Average anthocyanins recoveries (*n* = 3) with different solvent percentages. A different letter over a bar indicates a significant difference (*p*-value < 0.05).

Some authors have observed that percentages of methanol near 70% were more optimal for the extraction of anthocyanins, especially from plant materials [19,35,49]. Finally, higher levels of methanol in the solvent were assayed while the rest of optimal extraction conditions according to the BBD were applied, i.e., 70 °C, 20 mL of solvent per 0.5 g of sample and pH = 5. Figure 4 shows the recoveries obtained when using 50%, 60%, 70%, 80%, 90% and 100% MeOH as the extracting solvent. It can be seen that for MeOH values between 50 and 90% the anthocyanins recoveries did not present any significant differences. On the contrary, when 100% MeOH was employed, a lower recovery was achieved.

### 3.2. Optimum Extraction Time

**Figure 5:**
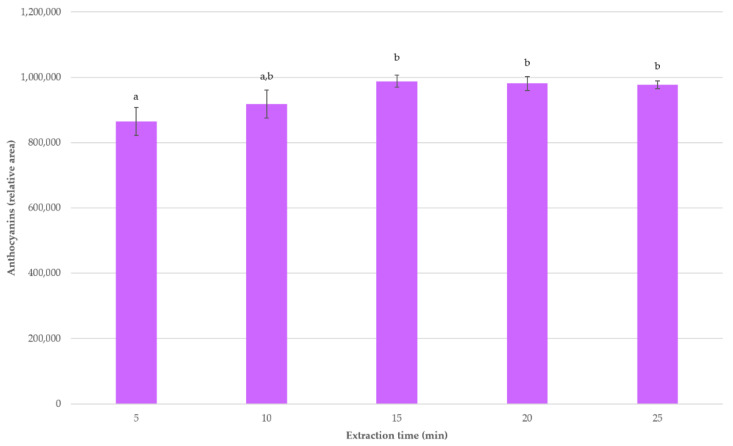
Average anthocyanins recoveries (*n* = 3) at different extraction times under the established UAE optimum conditions. A different letter over a bar indicates a significant difference (*p*-value < 0.05).

Once the optimum conditions for the extraction of anthocyanins from *E. australis* flowers were determined, a kinetics study of the extraction process was conducted. For that purpose, 0.5 g samples were extracted under the established optimum conditions using different extraction times that ranged from 5 to 25 min. The extractions were performed in triplicate for each extraction time. The average total anthocyanins extraction for each experimental time is displayed in Figure 5.

As it can be observed, the maximum amount of anthocyanins was extracted when the extraction time was 15 min, with a noticeable difference in relation to the 5 min extractions and no significant differences with regard to any other of the extraction times. This fact is in agreement with the extraction time required for the extraction of anthocyanins from other matrices [19,35,36]. Moreover, no decreasing trend in anthocyanins recoveries was observed when longer extraction times (over 15 min) were applied, which suggests that no anthocyanin degradation took place under such time conditions [50].

### 3.3. Precision Study

In order to determine the repeatability and intermediate precision of the optimized UAE method a number of extractions were performed under the optimum conditions (0.5 g samples extracted with 20 mL of solvent (50% MeOH:H_2_O) at pH 5 for 15 min at 70 °C).

For the repeatability study, 9 extractions were carried out on the same day. While for the intermediate precision study, 4 extractions were performed on each one of three consecutive days. All of the extractions were conducted according to the previously established optimum conditions. The average relative area and the residual relative standard deviation (RSD) have been summarize in Table 3.

The study results were all satisfactorily below 5%, with 3.31% and 3.52% for intra-day and inter-day RSD respectively, which confirms a high precision level of the optimized UAE methodology for the extraction of anthocyanins from *Erica* flowers.

### 3.4. Application of the Developed Method to Other Samples

**Figure 6:**
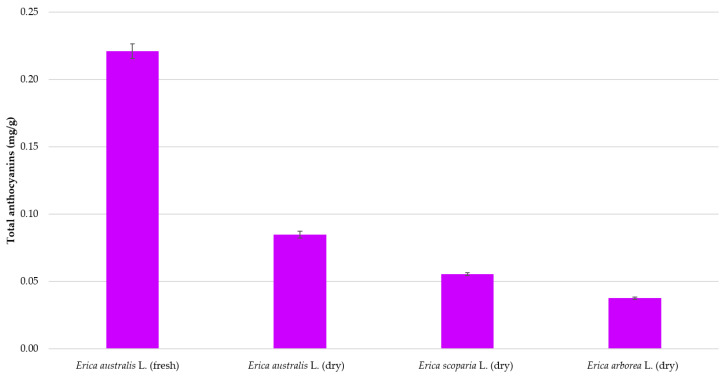
Total anthocyanins recovery from *Erica* genus flowers extracted under the established optimum conditions (*n* = 3).

Lastly, the optimized method was applied to flower samples from three different *Erica* species comprising dry *E. australis*, *E. arborea*, and *E. scoparia* and fresh *E. australis* (from Cadiz). The total anthocyanins recoveries have been presented in Figure 6 and based on them, it can be affirmed that the UAE methodology that has been optimized in this study can be successfully and reliably applied to the extraction of anthocyanins from the flowers of the *Erica* genus.

Let us mention that significant differences in anthocyanins content were detected between the studied samples according to their origin or variety, where fresh *E. australis* flowers from the Cadiz area exhibited the largest anthocyanins recoveries reaching up to 0.22 (mg/g), followed by dry *E. australis*. Cyanidin 3-*O*-glucoside has been the majority anthocyanin in all the samples analyzed. In view of the results, the use of fresh *Erica* flowers is advised.

## 4. Conclusions

In the present study, an ultrasound-assisted extraction methodology has been optimized to ensure the maximum recovery of anthocyanins from *E. australis* flowers. The percentage of methanol in the solvents, the extraction temperature, and the mass to solvent ratio have exhibited a significant influence on the total anthocyanins recoveries with *p*-values below 0.05. In addition, the effect caused by the interaction between the solvent composition and the extraction temperature has similarly proven to be determinant for the extraction results.

The optimum conditions for the maximum anthocyanins extraction were established at 0.5 g samples extracted using 20 mL of solvent (50% MeOH:H_2_O) with pH 5 and at 70 °C. Optimal extraction time was established at 15 min, since maximum recoveries were achieved and no significant differences could be noticed when longer times, up to 25 min, were used.

The repeatability and intermediate precision studies determined intra-day and inter-day RSD at 3.31% and 3.52% respectively, which confirms the satisfactory precision level of the optimized UAE methodology. Finally, the developed method has been applied to the dry flowers of three different *Erica* species (*E. australis*, *E. arborea*, and *E. scoparia*) and to the fresh flowers of *Erica australis* and its suitability for the extraction of anthocyanins from *Erica* matrices has been confirmed.

The results obtained in this study together with the well-known an numerous advantages associated to ultrasound-assisted methodologies, such as its rapid, uncomplicated and economic use and its scarce maintenance requirements, represent a substantial improvement in extraction techniques for numerous attractive matrices associated to health promoting properties or other uses such as those of *E. australis* flowers.

The authors consider the promising results obtained from this research to be a starting point for further research in a wide variety of fields. For example, the application of the UAE technique in the extraction of anthocyanins at an industrial level for the formation of enriched complexes or obtaining extracts that can be used in the food, pharmaceutical or cosmetic industry. In addition, these results lead to much more in-depth studies on the possible relationship between the bioactivity of anthocyanins and their bioavailability, as well as in the study of the content of anthocyanins in different *Erica* species, or the expression of anthocyanins in *Erica* species depending on the environmental conditions or external effects suffered by these plants.

## Figures and Tables

**Table 1:** Box-Behnken design experiment for the optimization of total anthocyanins extraction from *Erica australis* flowers.

Experiment	%MeOH	Temperature (°C)	pH	Ratio (mL)	Total Anthocyanins Measured (Relative Area)	Total Anthocyanins Predicted (Relative Area)	Relative Error in the Prediction (%)
1	25	10	5	10	337,455	389,145	15
2	50	40	5	10	639,247	647,347	1
3	50	40	5	20	757,508	775,737	2
4	25	40	5	15	590,048	563,405	5
5	25	70	5	10	573,794	551,805	4
6	25	40	3	20	660,597	640,939	3
7	25	40	7	10	505,854	494,633	2
8	50	40	7	15	718,199	718,545	0
9	50	10	5	15	725,040	648,277	11
10	25	70	3	15	657,311	643,549	2
11	25	40	7	20	616,314	608,983	1
12	0	40	3	15	349,368	365,311	5
13	25	70	7	15	603,419	621,473	3
14	0	40	5	10	278,416	276,197	1
15	0	70	5	15	411,697	457,587	11
16	25	40	3	10	532,038	508,509	4
17	25	10	3	15	461,176	458,929	0
18	50	70	5	15	720,040	727,837	1
19	25	10	5	20	451,491	489,735	8
20	25	10	7	15	405,624	435,173	7
21	0	40	7	15	348,784	320,695	8
22	50	40	3	15	675,303	719,761	7
23	0	10	5	15	204,944	166,227	19
24	25	40	5	15	572,485	563,405	2
25	25	40	5	15	527,052	563,405	7
26	25	70	5	20	733,466	697,995	5
27	0	40	5	20	386,912	394,587	2

**Table 2:** BBD-RSM analysis of the total anthocyanins recovery from *Erica australis* flowers.

Variable	Sum of Squares	*F*-Value	*p*-Value
%MeOH	4.24 × 10^11^	216.04	0.000
Temp	1.03 × 10^11^	52.72	0.000
pH	1.58 × 10^9^	0.80	0.387
Ratio	4.56 × 10^10^	23.23	0.000
Temp × Temp	6.94 × 10^9^	3.54	0.180
pH × pH	3.98 × 10^9^	2.03	0.849
Ratio × ratio	7.44 × 10^7^	0.04	0.843
%MeOH ×Temp	8.02 × 10^7^	0.04	0.034
%MeOH × pH	1.12 × 10^10^	5.71	0.632
%MeOH × ratio	4.73 × 10^8^	0.24	0.914
Temp × pH	2.38 × 10^7^	0.01	0.985
Temp × ratio	688,900	0.00	0.616
pH × ratio	5.21 × 10^8^	0.27	0.842

**Table 3:** Precision study on anthocyanins UAE from *Erica australis* flowers.

	Repeatability ^1^	Intermediate Precision ^2^
Average	966,332.07	1,008,543.09
SD *	32,003.30	35,475.37
RSD **	3.31	3.52

^1^ Repeatability (*n* = 9); ^2^ Intermediate precision (*n* = 12); * Standard deviation; ** Relative standard deviation.

## Data Availability

The data presented in this study is contained within the article.

## Sample Availability

Samples of *Erica* flowers are available from the authors.
